# The Concept of the Constructional Solution of the Working Section of a Robot for Harvesting Strawberries †

**DOI:** 10.3390/s21113933

**Published:** 2021-06-07

**Authors:** Sławomir Kurpaska, Andrzej Bielecki, Zygmunt Sobol, Marzena Bielecka, Magdalena Habrat, Piotr Śmigielski

**Affiliations:** 1 Faculty of Production and Power Engineering, University of Agriculture in Krakow, Mickiewicza Av. 21, 31-120 Krakow, Poland; zygmunt.sobol@urk.edu.pl; 2 Faculty of Electrical Engineering, Automation, Computer Science and Biomedical Engineering, AGH University of Science and Technology, Mickiewicza 30, 30-059 Kraków, Poland; bielecki@agh.edu.pl; 3 Faculty of Geology, Geophysics and Environmental Protection, AGH University of Science and Technology, Mickiewicza 30, 30-059 Kraków, Poland; bielecka@agh.edu.pl (M.B.); mhabrat@agh.edu.pl (M.H.); 4 Humtap Inc., Sarego Street 26/16, 31-047 Kraków, Poland; smigielski.piotr@gmail.com

**Keywords:** harvesting robot, strawberry, vision system

## Abstract

Strawberry fruits are products of high commercial and consumption value, and, at the same time, they are difficult to harvest due to their very low mechanical strength and difficulties in identifying them within the bush. Therefore, robots collecting strawberries should be equipped with four subsystems: a video object detection system, a collecting arm, a unit for the reception and possible packaging of the fruit, and a traction system unit. This paper presents a concept for the design and operation of the working section of a harvester for strawberry fruit crops grown in rows or beds, in open fields, and/or under cover. In principle, the working section of the combine should meet parameters comparable with those of manually harvested strawberries (efficiency, quality of harvested fruit) and minimise contamination in the harvested product. In order to meet these requirements, in the presented design concept, it was assumed that these activities would be performed during harvesting with the natural distribution of fruits within the strawberry bush, and the operation of the working head arm maneuvering in the vicinity of the picked fruit, the fruit receiving unit, and other obstacles was developed on the basis of image analysis, initially general, and in detail in the final phase. The paper also discusses the idea of a vision system in which the algorithm used has been positively tested to identify the shapes of objects, and due to the similarity of space, it can be successfully used for the correct location of strawberry fruit.

## 1. Introduction

The most important problems in crop production are labour-intensive operations that require human labour. Such operations include, inter alia, harvesting fruit or vegetables sensitive to mechanical damage, and combating weeds between plants in rows. This fact necessitates research on autonomous tractors, robotic platforms, and autonomous machines used in field work. Marinoudi et al. [1] presented the effects of using robots in agriculture, including short- and medium-term impacts on jobs, and technological inputs, and presented a framework of studies to assess the impact of robotic applications on the food production process. Pedersen et al. [2,3] state that in order to build a robotic solution, a system analysis should be carried out, which should include operational aspects, costs, and benefits of the designed machine. The designed robotic system should meet the following requirements: it should be low in weight, small in size, have a high level of intelligence, communication, and safety, and be effectively adaptable to a potential task [4]. Currently, robotic systems used in plant production are classified as devices used for weeding, sowing, detecting diseases and insects, monitoring plant development, chemical plant protection treatment, harvesting, and multifunctional systems [4]. The challenges faced in agricultural work are related to both the versatility and the specificity of the tasks performed. The most common issues are the assessment of the terrain [5,6], route planning [7,8], work safety, especially of people [9], and the work of teams of robots [10]. The most important factors in specific problems are detection and classification of crops or pests, and the architecture of crops. These problems relate to the design and operation of the vision system, navigation system in agricultural environments, and control systems for both the robotic platform and tools. Automated production is an important current trend applied in various production fields and, in the future, it will be necessary to also apply this to agriculture. As technologies gradually evolve, the use of robotics in agricultural production is gaining importance. Research on the design and implementation of robots in agricultural production is also an important factor in building a production environment for high-quality agricultural products. Harvesting is a repeatable activity, and one of the most labour-intensive in the crop production cycle. Taking into account the ripeness phase of fruits or vegetables, two types of robots are constructed for harvesting: ones which collect all vegetables (or fruit object by object) and ones for selective harvesting (only fruits that have reached so-called harvest ripeness are collected). The greatest challenge in the construction of robotic systems are units that carry out selective harvesting. This type of harvest is characterised by two parameters: the speed of performing a single operation, and the picking rate (i.e., the number of fruits successfully harvested from the number of fruits ready for harvest) [11]. Many of the selective harvesting robots currently being constructed focus on solutions for picking strawberry fruit [12,13]. Strawberry fruits are products of high commercial and consumption value, and, at the same time, they are difficult to harvest due to their very low mechanical strength and difficulties in identifying them within the bush. According to the available data, the consumption of strawberry fruit in the USA (whose strawberry production constitutes almost 20% of the world’s cultivated area) has increased fourfold in the last two decades [14]. However, the production of strawberries is highly dependent on human labour, and this labour cost is significant in the total structure, especially as regards harvesting [15]. To reduce production costs, improve product quality, and reduce dependence on human labour in the cultivation of soft fruit, several research groups are working on fruit-harvesting robots. For example, in the USA, nearly 70% of economic entities in the strawberry industry have invested in fully robotic strawberry harvest systems [16]. Robots which pick fruit and flowers are a topic that has been worked on in the field of agricultural robotics for over a dozen years. An important element of this type of robot is its vision system [17,18,19,20,21,22]. Efficient and reliable robotic strawberry picking has proved to be extremely difficult for several reasons. First of all, strawberries, which require delicate handling when picked, are easily damaged mechanically [23]. Second, strawberry harvesting requires highly selective procedures [24], because strawberries tend to ripen very unevenly, resulting in large variations in colour and size. In addition, strawberries need to be harvested systematically. An additional difficulty in harvesting is that strawberries grow in clusters, which makes it difficult to identify and select individual objects [15]. Based on an analysis of the technological process, strawberry picking robots should be equipped with four subsystems: a visual object detection system, an arm with a collecting head including an end effector, a unit for collecting and possible packaging of fruit, and the unit’s traction system. The fruit-picking working head is a critical component of this robotic system as it should allow for proper manipulation [20] and pick fruit in a gentle and effective way. An appropriate gripper design can significantly improve the stability and performance of the system [25]. Thus far, several types of working heads for picking strawberries have been developed: scissor-like devices [24], knives with a suction device [24], grippers with adjustable gripping force [23], jaw grippers with jaws controlled by flexible cables [26], and vacuum grippers [27]. De Preter et al. [28] presented the effects of the work of a strawberry-picking robot with a finger gripper installed. The gripper, on the contact surface with the strawberry, is equipped with a skeleton structure, thanks to which it minimises damage. A broad overview of the grippers used, together with a detailed description of the effects when using the vacuum gripper, is presented in [27]. As the position of the strawberry stem (collection point) is difficult to detect [29,30], especially within the bush habitat, scissor heads require a relatively complicated vision system solution. It is also easy to cut more than one petiole and accidentally destroy the green fruit of the strawberry plant. Pressure-controlled grippers are also difficult to use because they can easily damage the delicate fruit [24]. It seems advisable to use a vacuum gripper, which should be an integrated part of the collecting head (the head supplemented with a mechanical gripper). Such a solution results directly from the idea of the operation of the gripper: air flowing through the suction cup of the gripper facilitates access to the strawberry fruit.

In order to increase the efficiency of harvest, since the 1990s, research on equipment (robots) for fruit harvesting has been conducted in the Netherlands and Japan, and as a result, commercial prototypes have been developed [24,31,32,33,34,35,36,37]. Many problems related to the harvesting of soft fruits are still unsolved. It seems that the reason for this is the little-known working environment of the collecting heads. The first robot for harvesting strawberries grown in hanging benches was developed by Japanese scientists in 2002 [24]. The robot can harvest one fruit in 11.3 s and the success rate is 41.3%. Chinese scientists have developed a robot that performs strawberry-planting or fruit-harvesting operations after installing the appropriate working heads [38,39,40,41].

Experiments conducted in a real environment under cover in the cultivation of strawberries in the hanging bench system with the use of a jaw gripper with jaws controlled by flexible rods indicate that the time range in the continuous picking cycle of single strawberry fruits, taking into account all procedures, is from 7.5 to 10.6 s. The robot discussed here is capable of picking single strawberry fruits with a success rate of up to 96.8%. However, in field conditions, where the individual fruits are selected from clusters, the average success rate is from 53.6% to 59.0% (the latter if mechanically damaged fruits are included). The low value of the success rate while collecting fruit from strawberry fruit clusters in the operational mode results from the difficulties faced by the algorithm in detecting objects and by the gripper that harvests the fruit [26].

Recent trends in the design of strawberry fruit-picking robots include: (a) attempts to design and develop a work cycle and a picking gripper that do not subject the fruit to stress, causing it to be damaged, and with a drive that is highly tolerant to position errors and which can reduce the time it takes to place the fruit in the storage container; (b) attempts to tightly integrate all the strawberry fruit-picking subsystems, enabling the robot to pick continuously from a moving mobile platform; (c) development of a vision system that will identify failures of a single subsystem or the entire system, useful for further system improvement [26,42].

In light of the above comments, taking into account the huge demand for a harvester for harvesting strawberries, this paper presents a concept for the construction of the harvester’s working section dedicated to harvesting strawberries from open field and/or under cover crops, carried out in rows or beds. Additionally, the paper presents the structure of a robot vision system, the analysis environment, the creation of two- and three-dimensional representations of a single object, and the creation of a representation of the spatial relationships between objects.

## 2. Design Concept of the Working Section of the Strawberry Fruit Harvester

As has been shown, the construction of the harvester for mechanised strawberry harvesting is an extremely important problem (having application significance). The construction should take into account: the existing constructions of working units that automatically harvest the fruit, the quality of the harvested crop, and operational and economic aspects of the harvest. Hence, the concept of an alternative solution (compared to the existing solution) is presented, in terms of mechanics, in which the aim is to be able to harvest strawberries in rows or beds, in the field and/or under covers. The following functions were assumed in the analysis:

F1—harvesting, picking the fruit, and its transfer to the transport unit,

F2—exposing the fruits located in the inner part of the bush,

F3—manoeuvring the collecting head,

F4—fruit transport from the picking unit to the destination,

F5—displacement of the working section relative to the soil surface.

The following requirements were set for the constructed working section within the mechanical system (including the specified F functions):

- the working section can be used in autonomous or non-autonomous harvesters;
- the work (pick) efficiency of the working section will be comparable or higher in relation to the harvesting efficiency of a human (approx. 7 kg· h^−1^);
- the quality of picked fruit, determined by limiting the occurrence of mechanical damage, will be high;
- contamination of the picked fruit crop with parts of the plant or substrate, e.g., broken leaves, unripe fruit, used mulch, or soil, will be limited.

### Searching for Optimal Solutions Using the Morphological Table Method

Morphological analysis is one of the most popular techniques of creative thinking, and it belongs to the so-called combinatorial methods. Is mainly used to solve problem setpoints, and in particular for decision making. The problem-solving method consists in presenting a number of possible solutions and considering possible combinations together to determine the optimal solution (Table 1).

The assessment of the construction of individual solutions of the working units of the section for strawberry fruit harvesting was carried out within four groups of criteria. Weights were of the greatest importance for: the group of criteria for the assessment of design features and assessment of work technology. These groups were assigned 40 points each with a 100-point weight range. The weights were evenly distributed between the periodic service and economic evaluation criteria (Table 2). The assessment of individual design solutions was carried out within all assessment criteria, issuing an appropriate assessment for a given solution (Table 3). The best design solution for individual variants of solutions, teams performing appropriate functions, was determined by calculating the sum of the products of weights and ratings, while finding the highest value of these relationships.

With the evaluations, individual variants of design solutions will be based on the use of the following mechanisms:

- collecting head—a gripping unit with a chamber capacity greater than the volume of the fruit, assisted by a vacuum gripper,
- fruit detection support unit—finger comb that works periodically,
- manipulator arm assembly—manipulator arm assembly equipped with articulated and linear displacement mechanisms,
- conveyor unit—belt conveyor for transporting fruit,
- running gear—a tracked traction unit telescopically connected to the frame.

Referring to the obtained test results, for the assessments carried out, mainly on the collecting head, it should be stated that the new design solution of the collecting head—a gripping unit with a chamber capacity greater than the volume of the fruit, assisted by a vacuum gripper (suction cup), received a higher rating (764 points) in comparison with assessments of known solutions [23,24,26]. The use of a fruit detection support unit in the constructed working section (a similar solution has not been provided in any of the structures presented to date) should contribute to increasing the harvesting efficiency (measured by the ratio of the number of harvested fruits to the number of all fruits technologically suitable for harvesting) and greater efficiency of the vision system’s performance. Based on the obtained assessments of the two basic mechanical units (the collecting head and the fruit detection support unit) of the working section for strawberry fruit harvesting, it should be assumed that its output (quantitatively and qualitatively) will be at least the same as or higher than already known constructions.

A more detailed description of the concept of the structure and its operation, the working section for harvesting strawberry fruits, is provided later in the work.

**Figure 1:**
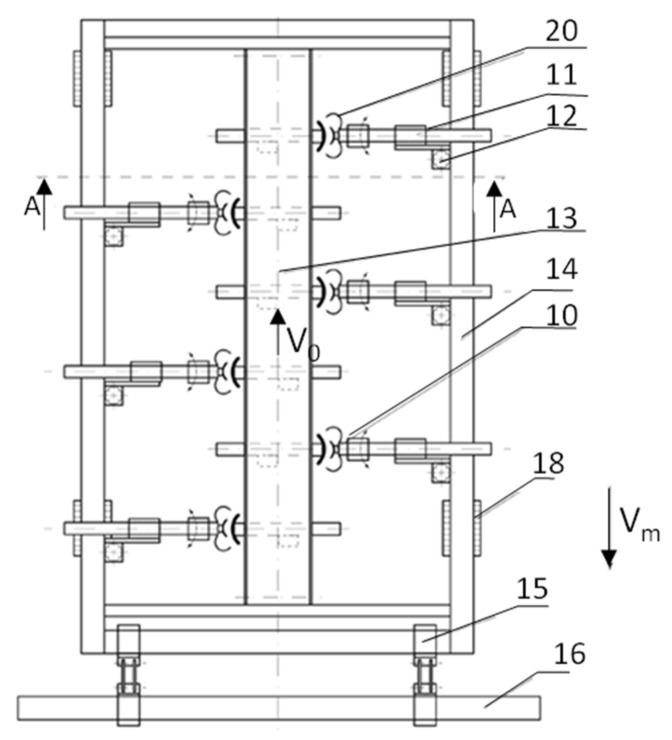
The working section of the harvester for harvesting strawberries, in a schematic view from above: 10—working head; 11—head arm longitudinal feed mechanism; 12—rotation mechanism in the horizontal plane; 13—belt fruit conveyor; 14—autonomous frame; 15—suspension system; 16—machine main frame; 18—tracked running gear; 20—tilting jaws; (Vm—forward speed of the combine). Source: Reprinted with permission from ref. [43]. Copyright in proceeding Copyright Sobol Z., Baran D., Nawara P., Kurpaska S.

The concept of the construction solution of the combine is shown schematically in Figure 1. A patent application for the protection right of a utility model, entitled Working section of a harvester for harvesting strawberry fruit, application number: W.128367 [43], has been submitted.

**Figure 2:**
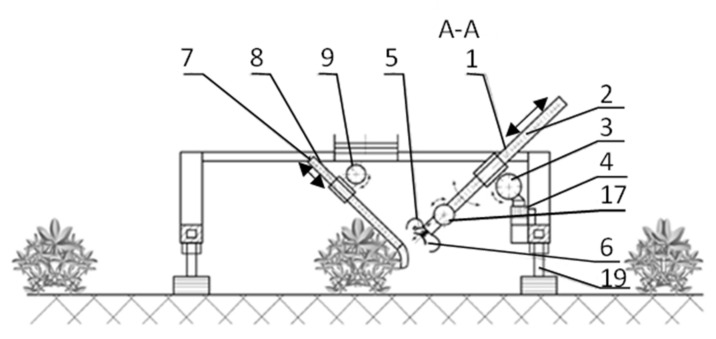
The working section of the harvester for harvesting strawberries, in a schematic view in section A—A: 1—working head arm; 2—rack of the longitudinal feed mechanism; 3—rotation mechanism in the vertical plane; 4—head arm rotation mechanism in the horizontal plane; 5—camera identifying the picked fruit; 6—pull-out suction cup; 7—comber arm; 8—longitudinal feed mechanism; 9—rotation mechanism in the vertical plane; 17—rotation mechanism in the vertical plane; 19—telescopic shock-absorbing suspension system. Source: Reprinted with permission from ref. [43]. Source: Reprinted with permission from ref. [43]. Copyright in proceeding Copyright Sobol Z., Baran D., Nawara P., Kurpaska S.

Elements of the pneumatic system of the working section are supplied with air from a vacuum pump mounted on the main frame of the combine or on the section frame, servicing the mechanisms of a given section. The pump drives all working sections in the combine. The combine working section has an autonomous frame (14), on which the mechanisms responsible for the harvesting process and traction functions are mounted. The section is connected to the combine’s main frame (16) by means of a suspension system (15), the structure of which is an articulated parallelogram. The use of this type of suspension ensures that the section frame is arranged parallel to the ground, regardless of its temporary position determined by the shape of the soil surface. Figure 2 (which is an extension of Figure 1) shows a diagram of the combine’s working section.

The traction system consists of a tracked chassis (18) and a telescopic shock-absorbing suspension system (19). The traction system is a four-point support of the combine’s working section against the ground. The use of crawler units and a telescopic suspension system is aimed at minimising the transmission of vibrations to the section frame. The telescopic and shock-absorbing suspension system (19) should include an adjustment of the position of the section frame in relation to the ground, enabling the harvesting of fruit either from crops grown on flat or raised beds. The working section is equipped with six or more working units (with head and arm control mechanisms) harvesting fruits. The number of working units depends on the maximum number of fruits harvested from one strawberry bush in one harvesting cycle. The unit directly performing the harvesting of the fruit is the working head (10) attached to the head arm (1). The head arm is divided into two sections, the longer one covering approx. 80% of the length of the entire arm, on which the rack of the arm longitudinal feed mechanism (2) is used, and the shorter one, to which the working head (10) is attached. The connection of both parts of the arm (1) is realised by the head arm rotation mechanism in the vertical plane (17). The second head arm rotation mechanism in the vertical plane (3) acts as a subassembly of the assembly connecting the arm with the section frame (14). The rotation of the head arm (1) in the horizontal plane is performed by the head arm rotation mechanism (4/12). Additional mechanisms participating in the fruit-picking process are combers (finger structures) attached to the arms (7). The comber interacts with the task of the working unit (head together with the arm and control mechanisms) and, similarly to the working unit, is controlled by a microprocessor controller, and the scope of control will depend on the analysis of the object image and the location of the collecting unit. The comb handler will tilt part of the strawberry bush to reveal the ripe fruit that is the subject of the harvesting process. The combers provide harvesting support for the third to the last working units. The combing mechanism is controlled longitudinally by the longitudinal feed mechanism of the comb (8), and in the vertical plane by the rotation mechanism (9). Longitudinal control of the arms of the head and combers is performed with the use of rack and pinion mechanisms, and the rotation of the arms with the use of toothed gears. These mechanisms are powered by electric motors. The working head (10) comprises tilting jaws, and its capacity is shaped in such a way that it will fully, in a non-invasive manner, contain the harvested fruit. In the central part of the bottom of the head, a pneumatic suction cup (6) is used, the task of which is to collect the ripe fruit, and initially move it (position and extract it from the bush mass) to a position in which the head harvesting the ripe fruit does not gather contamination with the fruit (torn leaves, unripe fruit, pieces of litter, or pieces of soil). The suction cup (6), depending on the existing conditions (fruit location), adjusts its position, extending from the head along the axis of the head arm (1). After the fruit is grasped by the suction cup, the suction cup and the head move in such a way (retracting the attachment and moving the head towards the fruit) as to place the fruit inside the head non-invasively. Depending on the purpose of harvesting (for consumption or processing purposes), the head will be equipped with a unit that cuts off the harvested fruit from the petiole. Consumer fruit which is not intended for processing should contain a stalk. In order to detail the image used to control the working mechanisms of the working (collecting) unit and the comb, a camera identifying the collected object (5) is provided at the bottom of the working head (10). The fruit collected by the working team is transferred to the fruit conveyor belt (13). The presented solution from the mechanical point of view has been thoroughly analysed, and the mechanisms used to meet the criteria that can fulfil the designated target functions related to operations performed with the technology of harvesting (obtaining) strawberry fruit (an application for a protection right was obtained). The applied mechanisms, as well as their configuration, in the construction concept, have been correctly adopted and bear the hallmarks of innovative solutions. The innovative solutions include at least two innovations: (a) the use of a sweeping unit, the operation of which will be activated after receiving a signal from the vision system (no technologically useful fruit for harvesting in the field of view of the camera in a given growing area) and (b) the construction of a mechanical gripper whose work is assisted by a pneumatic system (the use of a movable suction cup in the middle part of the gripper, the task of which is to extract (extract) the fruit from the inner part of the bush habit, to a position where the gripping mechanism can obtain the fruit without damaging it).

## 3. Hardware and IT System

**Figure 3:**
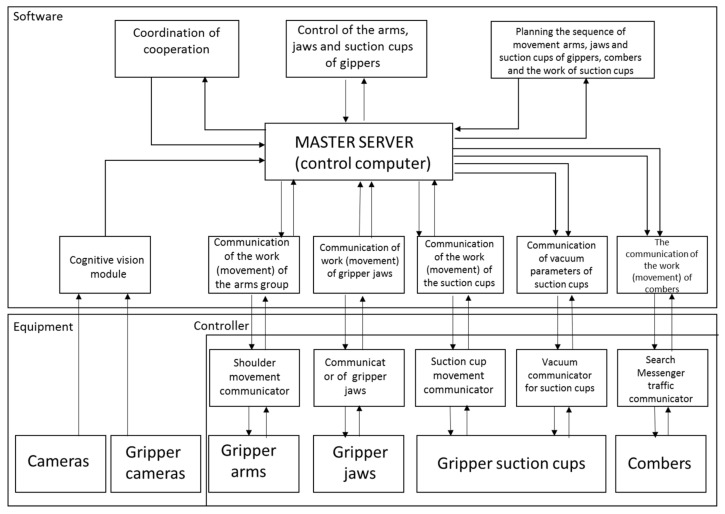
Functional diagram of the control system for the working section of the strawberry-picking robot.

The architecture of the hardware and information system of the robot’s working section is shown in Figure 3.

The working section is fully automated and consists of software modules and working units with drivers. The hardware modules are responsible for analysis (main server), image acquisition, and the work of the arms, jaws, suction cups, and combs, as well as the negative pressure of the suction cups. The layer of device control and data aggregation contains modules responsible for controlling the work of sensors, manipulators of the robot’s working section, and for the processing and fusion of the data provided by them. The robot system designer can use existing solutions to process and fuse sensor data, plan routes, and avoid obstacles for working arms with collecting heads and cleaners. In this area, the robot system designer can use any existing solutions or implement their own algorithms.

## 4. Cognitive Vision System of the Robot

The system used will be fixed to the machine frame (Figure 1 and Figure 2) and will be an integral part of the camera identifying the picked fruit and pull-out suction cup. Its vision system should perform two mutually independent tasks. First of all, it should ensure navigation in the environment on the basis of the algorithm presented by Bielecki and Śmigelski [44]. Precise determination of the location of the fruit outside the plant and inside it, i.e., fruit obscured by the shoots of the plant and its leaves, is the second task. Furthermore, the analysis of its ripeness phase will have to be done as well as determining whether it is rotten, mouldy, or mechanically damaged. As a consequence, the system will make a decision whether the fruit is suitable for picking or should be discarded due to damage or disease. Systems of this type have been constructed in the prototype form for apples [37,45], cucumbers [36,46], tomatoes [47], and peppers [48]. However, automatic strawberry harvesting, also analysed for a strawberry agricultural harvester, generates additional specific problems [24,26,49]. Strawberries, due to the texture of the fruit surface, have a lower light reflectance, which makes them more difficult to detect than apples, cucumbers, tomatoes, or peppers. In addition, strawberries are more hidden within the plant. Therefore, in the robot prototypes used, the localisation efficiency coefficient of both the strawberries and their stalks is relatively low—e.g., the fruit localisation efficiency is less than 54% [26] and, in the case of the peduncle, it is successfully localised only in about 60% of cases [24].

(a) As has been mentioned, strawberry visual detection and analysis has its own specifics. The module is planned to realise two tasks:
(b) Detection and analysis of the fruit quality on the basis of the colour and texture analysis;
(c) Analysis of mechanical damage on the basis of the fruit contour analysis.

**Figure 4:**
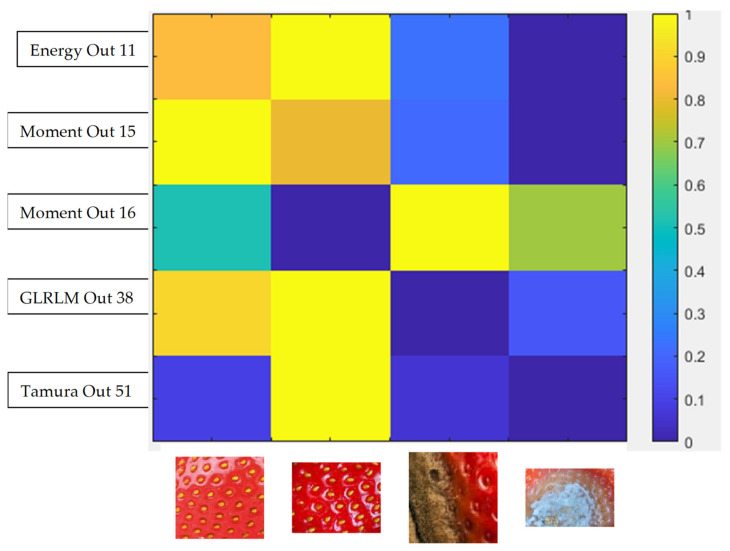
Visualisation of selected texture parameters for a ripe, very ripe, rotten, and mouldy fruit.

**Figure 5:**
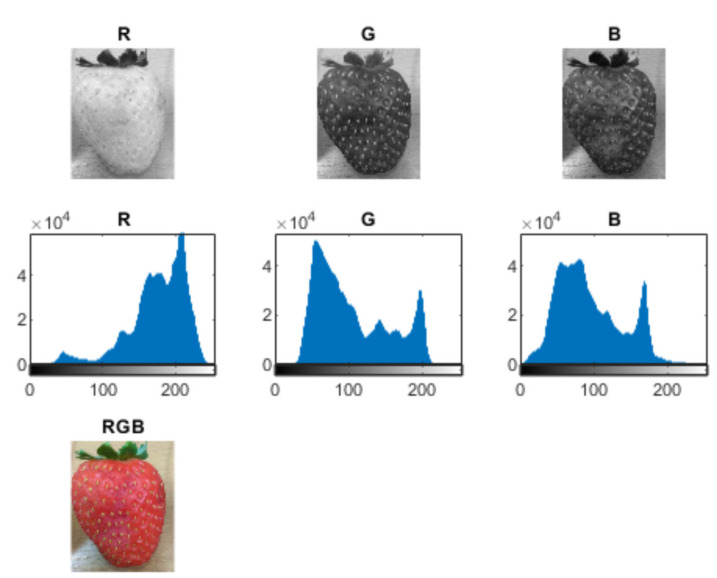
The brightness analysis—a ripe, healthy strawberry.

**Figure 6:**
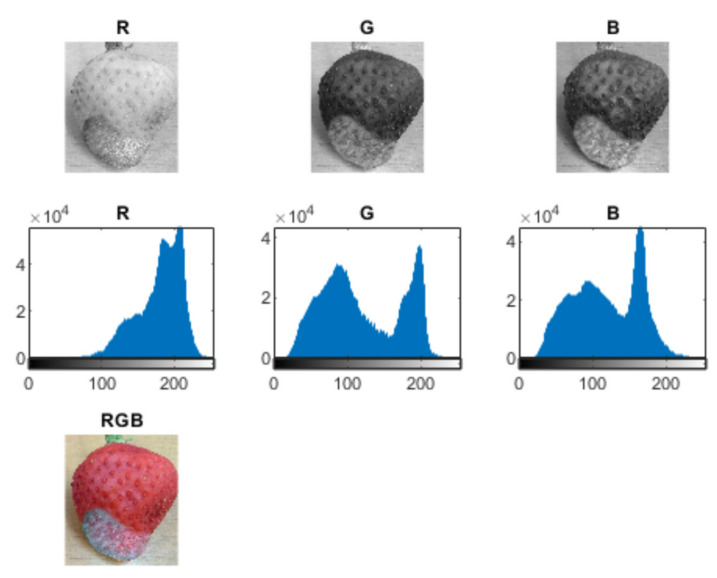
The brightness analysis—mouldy fruit.

The colour and texture analysis can be performed by adapting algorithms worked out for the analysis of geological samples—those by Habrat and Młynarczuk [50], Habrat and Młynarczuk [51]. Sample images of identified strawberry fruit (with varying degrees of damage) were examined. These images were represented in RGB space. For each image, the values of sample texture parameters were determined for grey images (so images were converted from RGB to greyscale). Approximately 50 different parameters from different parameter groups were analysed. The basic statistical parameters of grey levels include volume, coefficient of variation, standard deviation, skewness, energy, and entropy. Furthermore, histogram sums and differences, 1st and 2nd order moments, and grey-level co-occurrence matrix (GLCM) transformation parameters were considered (i.e., contrast, correlation, homogeneity, the inverse of difference moment, entropy, and difference sum and variance). The features of the grey-level difference matrix (GLDM) and grey-level run-length matrix (GLRLM) transformations and popular Tamura features were also evaluated. The results were normalised to an interval of 0 (low value) and 1 (high value). Feature extraction results are within in a feature vector that can be further analysed [50,51] with respect to the texture separation ability of fruit images with different degrees of damage. The effectiveness of the mentioned parameters varies, and a preliminary evaluation allows the selection of those parameters that have the ability to differentiate. Some texture parameters differentiate ripe, rotten, and mouldy fruits—see Figure 4. Furthermore, analysis of colour channels also shows differentiation depending on the existence of mould—see Figure 5 and Figure 6. There is a marked increase in high brightness in G and B channels for a mouldy fruit.

**Figure 7:**
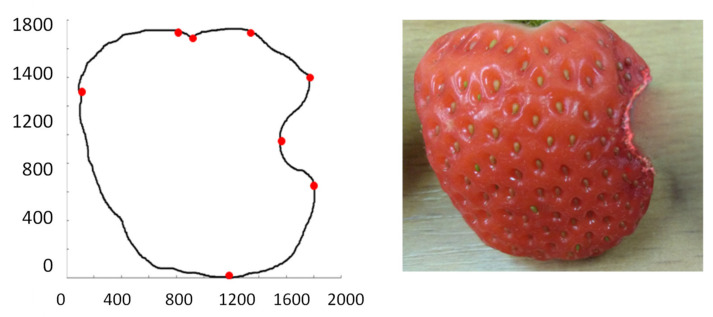
A fruit demaged on the inside. Its contour is described by the sinquad 14.41.14.43.34.43.32.21.

**Figure 8:**
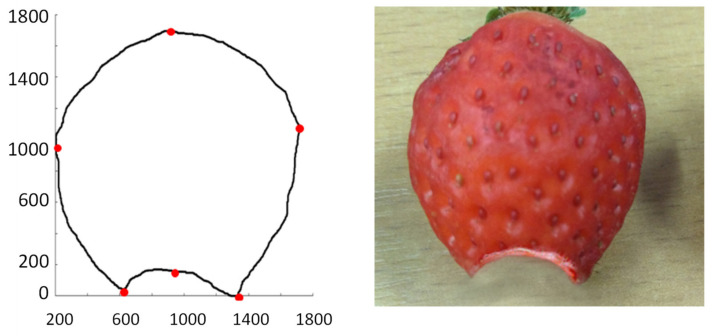
A fruit demaged on the inside. Its contour is described by the sinquad 14.43.32.23.32.21.

The fruit that is mechanically damaged, for example, when gnawed by a snail, may only have a changed shape without changing its colour—see Figure 6, Figure 7 and Figure 8.

**Figure 9:**
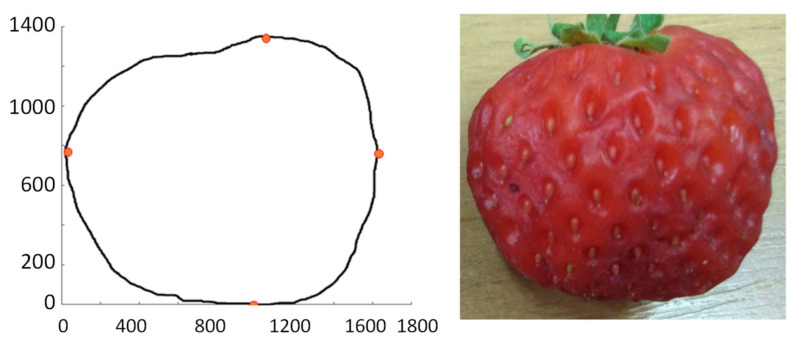
An example of undamaged fruit. Its contour is described by the sinquad 14.43.32.21.

**Figure 10:**
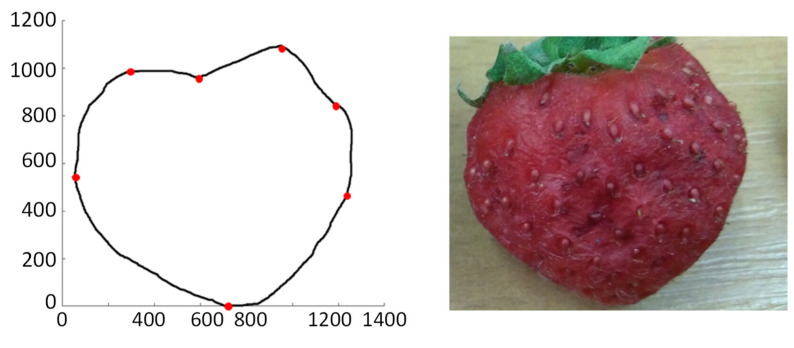
An example of undamaged fruit. Its contour is described by the sinquad 14.41.14.43.32.21.

Using the structural–syntactic contour analysis described in Bielecka et al. [52], Bielecka et al. [53], and, first of all, Bielecka [54], the contour analysis of undamaged and damaged strawberries was carried out. Structural–syntactic analysis of the contour consists in dividing the examined contour into primitives. The proposed primitives are fragments of curves forming a 16-element set. Each of the primitives carries information about the angle of inclination, curvature, and increment for x and y coordinates. A characteristic feature of a single sinquad is the information on to which quadrant of the coordinate system it belongs. As a result of the analysis of healthy strawberries, the descriptions of the following outlines were obtained: 14.43.32.21 and 14.41.14.43.32.21. The pattern shapes of undamaged strawberries are in Figure 9 and Figure 10. The contour understudy in Figure 10 below has been divided into four sinquads. The red dots indicate the beginning and end of subsequent sinquads. However, the shape of the strawberries is regular and therefore the number of possible patterns is small. In the process of recognising the shape of the strawberry, the obtained contour is compared to the tested patterns. An uncharacteristic indentation in any sinquads indicates damage to the fruit. The descriptions of undamaged and damaged fruit are in Table 4.

The proposed method allows for describing the shape of objects of various sizes but that similar in shape. At the same time, the shape of the strawberry is regular, which means that there will be only a few patterns. This means that identifying strawberries with damaged contours should be effective.

To sum up, the preliminary analysis shows that the methods based on the analysis of texture and colour as well as the shape of the fruit contour are effective in distinguishing ripe and healthy strawberries from damaged, rotten, and mouldy fruit.

## 5. The Described System and An Autonomous Robot

The robot will run in greenhouses. Its vision system should ensure collision-free navigation in the environment. The cognitive vision system in the robot realising the said task is planned to consist of three independent modules:

1 Scene analysis module;
2 Anti-collision module detecting humans and animals (similar as described earlier);
3 Fruit detection and quality rating module.

**Figure 11:**
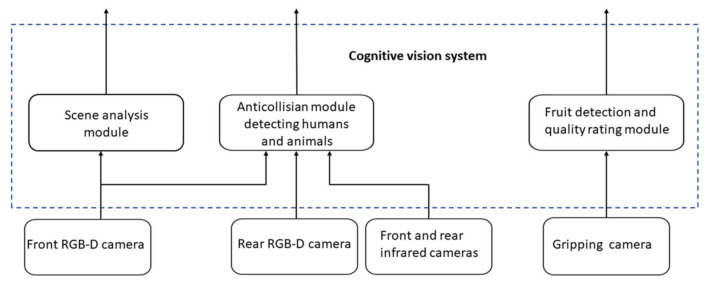
The structure of the cognitive vision system.

The functional scheme of the whole system is presented in Figure 11.

### 5.1. Scene Analysis Module

The scene analysis module can be adapted from the vision system worked out for autonomous aerial agents [45,55]. Let us recall briefly the crucial components and functionalities of the said system. The algorithm creates a three-dimensional representation of a human-made environment that consists of artificial objects such as structures which are composed of some number of basic construction elements: rectangular prisms and pyramids. The algorithm consists of the modules that realise the following functionalities:

(a) Creation of two-dimensional projections of single objects;
(b) Creation of three-dimensional representations of single objects;
(c) Creation of representations of spatial relations among the objects;
(d) Constructing a three-dimensional representation of the scene;
(e) Scene analysis, including the environment “understanding” in the context of the realised task.

Since the greenhouse environment is a human-made environment, the worked-out algorithm [44,55] can be almost strictly adapted. The only modification is connected to the fact that the harvesting robot will operate inside the bounded area. This fact can be, however, easily encoded by using the tools for the representation of objects in the system.

### 5.2. Anti-Collision Module Detecting Humans and Animals

Each autonomous robot that operates in an area in which living creatures—humans and animals—can unexpectedly emerge in the robot operating area has to be equipped with a system that enables the robot to avoid collisions with humans and animals. The module that enables the robot to detect living creatures that suddenly appear in the ro-bot’s field of view is the most important element of such a system. Furthermore, the system has to work on-line, thus the detection must be quick. The image from the infrared camera is a good starting point for such detection, because the temperature of living creatures is significantly different from the ambient temperature. This solution is a standard one. The robot is intended to be equipped with front and rear infrared cameras. In the case of using a set of autonomous robots in a greenhouse that perform the function of a target (harvesting strawberries), it will be necessary to apply the anticipatory network theory which should optimise the adopted performance criterion of a single robot. This issue was analysed, among others, in [56], in which the author specified two criteria in the organisation of the work of a team of robots: (a) recognition of time hierarchies by swarm vehicles and (b) definition of behavioural strategies that ensure the best achievement of a common goal. In another work, the authors [57] developed a simulation model of the operation of a swarm of autonomous robots, which assumed cooperation in the field of supervision and mitigation of threats adapted to environmental conditions.

## 6. Summary

The presented concept of construction should contribute to the improvement of strawberry fruit harvesting efficiency, reduce the generation of mechanical damage, and reduce crop contamination with parts of plants or the substrate by using the following units:

- combers, the effect of which will be to expose (reveal) fruits located in the inner parts of the bush;
- suction cups enabling the extraction of fruit from the bush habitat to such a position that the fruit can be picked up (picked) non-invasively by the jaws of the head;
- vision system identification sensors in the heads, which will contribute to the improvement of the effectiveness of head manipulation in the fruit grasping operation, and thus shorten the operation time.

The modular (sectional) construction of the robot will allow the adjustment of its construction parameters to the area of cultivation. The described assumptions of the vision system in autonomous robots should also give a positive effect in the form of automatic strawberry fruit harvesting.

The fruit identification algorithm presented in the work, together with the colour analysis, should also give positive results, because it was tested for an air agent which, similarly to the combine presented in this paper, identified objects from a certain distance. The discussed algorithm was tested in a vision system as a contour classifier used for the analysis of bone shape in X-ray images. When comparing the conditions of the fruit against the leaves and shoots of strawberry plants, it seems that such an analogy is highly appropriate.

## Figures and Tables

**Table 1:** Morphological table: optimal solutions for the construction of working units of the section for harvesting strawberry fruit in field crops and under covers.

Features of the Technical System	Variants of Solutions (Short Description)				
	I	II	III	IV	V
F1—collecting head	Scissor unit [24]	Gripper and finger unit with adjustable gripping force [23]	A gripping unit with a chamber capacity greater than that of the fruit, controlled by lines [26]	Suction cup and suction cup working with the knife [24]	Gripping unit with a chamber capacity greater than the fruit volume, assisted by a vacuum gripper
					xxx
F2—fruit detection unit	The system of nozzles working under positive pressure	A system of nozzles working under negative pressure	A finger comb that works cyclically	Rotary comb	----
			xxx		
F3—manipulator arm assembly	Assembly equipped with articulated mechanisms only	The unit is equipped with articulated and linear displacement mechanisms	---	---	---
		xxx			
F4—conveyor unit	Scraper conveyor	Conveyor belt	Screw conveyor	---	---
		xxx			
F5—running gear	A wheeled trainset rigidly connected to the frame	Tracked traction unit rigidly connected to the frame	A wheeled trainset unit telescoped to the frame	Tracked traction unit telescopically connected to the frame	---
				xxx	

xxx—the best design solution.

**Table 2:** Weights in the criterion assessment of the analyzed solutions.

Criteria for evaluation of design features:	40
1. Simplicity of construction;	8
2. The compactness of the structure;	12
3. Work reliability, taking into account the influence of the working environment;	10
4. Reliability.	10
Work technology assessment criteria:	40
1. Speed of the performed operation (vibration damping *);	10
2. Not generating mechanical damage to the fruit crop;	13
3. Not generating contamination of the fruit yield;	10
4. Low requirements for cooperation with the vision system.	7
Periodic maintenance evaluation criteria:	10
1. Ease of repair;	5
2. Self-diagnosis possible.	5
Economic evaluation criteria:	10
1. Low operating costs;	5
2. Low repair costs.	5

*—applies to the suspension system of the traction unit.

**Table 3:** Assessment of individual design solutions of variants of working teams, in relation to the adopted evaluation criteria, strawberry fruit-harvesting section.

Specification			Criteria for Evaluating Design Features				Work Technology Assessment Criteria				Periodic Maintenance Evaluation Criteria		Economic Evaluation Criteria		The Sum of the Products of Weights and Grades
Nr Criteria			1	2	3	4	1	2	3	4	1	2	1	2	
Scales			8	12	10	10	10	13	10	10	5	5	5	5	
Assessment of solutions	F1	I	7	10	5	8	5	8	4	3	4	4	4	4	610
		II	3	8	4	7	6	5	4	6	4	4	4	4	535
		III	6	7	4	8	5	6	5	5	4	4	4	4	560
		IV	6	8	6	8	7	9	6	7	4	4	4	4	681
		V	5	7	9	8	8	10	10	9	3	4	4	3	764
	F2	I	6	8	7	8	8	9	3	8	4	4	2	3	666
		II	6	8	5	5	5	9	5	8	4	4	2	3	606
		III	5	9	8	8	7	8	8	7	3	4	4	2	697
		IV	5	7	6	6	7	5	6	8	3	4	4	3	589
	F3	I	5	8	9	7	6	10	8	7	3	4	4	3	706
		II	5	7	9	7	9	10	8	8	4	4	4	4	744
	F4	I	5	9	8	8	7	5	8	9	4	4	4	4	693
		II	7	9	8	8	7	10	9	9	3	4	4	3	774
		III	6	9	8	8	7	5	8	9	4	4	4	4	701
	F5	I	7	8	5	7	3	4	6	7	5	4	4	4	569
		II	6	7	6	7	4	5	5	7	4	4	3	3	557
		III	6	6	6	7	6	6	6	7	4	4	4	3	598
		IV	5	6	9	8	9	7	5	7	3	4	3	3	648

**Table 4:** The structural description of the contour of undamaged and damaged fruits by using sinquads.

No.	Undamaged Strawberry	Damaged Strawberry
1.	14.43.32.21	14.43.32.23.32.21
2.	14.41.14.43.32.21	14.41.14.43.34.43.32.21

## Data Availability

Not applicable.
